# COVID-19 mRNA Vaccine Safety Among Children Aged 6 Months–5 Years — United States, June 18, 2022–August 21, 2022

**DOI:** 10.15585/mmwr.mm7135a3

**Published:** 2022-09-02

**Authors:** Anne M. Hause, Paige Marquez, Bicheng Zhang, Tanya R. Myers, Julianne Gee, John R. Su, Casey Parker, Deborah Thompson, Sarada S. Panchanathan, Tom T. Shimabukuro, David K. Shay

**Affiliations:** ^1^CDC COVID-19 Emergency Response Team; ^2^Food and Drug Administration, Silver Spring, Maryland.

On June 17, 2022, the Food and Drug Administration (FDA) amended the Emergency Use Authorization (EUA) for mRNA COVID-19 vaccines to include children aged 6 months–4 years for receipt of BNT162b2 (Pfizer-BioNTech) (administered as 3 doses, 3 *μ*g [0.2 mL] each) and children aged 6 months–5 years for receipt of mRNA-1273 (Moderna) (administered as 2 doses, 25 *μg* [0.25 mL] each) (*1*,*2*). In preauthorization clinical trials, the Pfizer-BioNTech vaccine was administered to 3,013 children aged 6 months–4 years (*3*) and the Moderna vaccine was administered to 5,011 children aged 6 months–5 years (*4*). Most adverse events reported in these trials were mild to moderate in severity and no serious vaccine-related adverse events were reported. To characterize postauthorization safety of COVID-19 vaccine primary series among young children, CDC reviewed adverse events and health impacts after receipt of Pfizer-BioNTech and Moderna vaccines that were reported to v-safe, a voluntary smartphone-based U.S. safety surveillance system established by CDC to monitor adverse events after COVID-19 vaccination (https://vsafe.cdc.gov/en/), and the Vaccine Adverse Event Reporting System (VAERS), a U.S. passive vaccine safety surveillance system managed by CDC and FDA. During June 18–August 21, 2022, approximately 599,457children aged 6 months–4 years received the Pfizer-BioNTech vaccine and 440,773 aged 6 months–5 years received the Moderna vaccine*; approximately 23,266 children were enrolled in v-safe after mRNA COVID-19 vaccination. The most frequent systemic reactions reported to v-safe after receipt of Pfizer-BioNTech or Moderna vaccines were irritability or crying among approximately one half of children aged 6 months–2 years. Among children aged ≥3 years, systemic reactions after vaccination were less frequently reported; injection site pain was the most frequently reported reaction among these older children. VAERS received a total of 1,017 reports of adverse events after Pfizer-BioNTech or Moderna vaccination among children aged 6 months–4 years and children aged 6 months–5 years; 998 (98.1%) events were classified as nonserious and 19 (1.9%) as serious. No reports of myocarditis after vaccination were reported. These initial safety findings are similar to those from preauthorization clinical trials (*3*,*4*). Health care providers and parents of young children should be aware that local and systemic reactions are expected after vaccination with Pfizer-BioNTech or Moderna vaccine and that serious adverse events are rare.

On June 20, 2022, v-safe was modified to allow parents and guardians to enroll children aged 6 months–4 years after any mRNA COVID-19 vaccine dose. Text message reminders are sent to parents or guardians to complete online health surveys for their child.^†^ Health surveys sent in the first postvaccination week include questions about local injection site and systemic reactions (i.e., mild, moderate, or severe) and health impacts.^§^ Specific questions were included for children aged 6 months–2 years who might not be able to describe reactions or who might experience reactions that are different from those experienced by children aged ≥3 years.^¶^ CDC’s v-safe call center contacts registrants who indicate that medical care was received after vaccination and encourages completion of a VAERS report.

VAERS is a national passive vaccine safety surveillance system managed by CDC and FDA that monitors adverse events after vaccination (*5*). VAERS accepts reports of postvaccination adverse events from health care providers, vaccine manufacturers, and members of the public.** Signs, symptoms, and diagnostic findings in VAERS reports are assigned Medical Dictionary for Regulatory Activities preferred terms (MedDRA PTs) by VAERS staff.^††^ Reports of serious events to VAERS^§§^ were reviewed by CDC and FDA physicians to form a consensus clinical impression based on available data. Using selected MedDRA PTs, a search was performed to identify possible cases of myocarditis, a rare adverse event that has been associated with mRNA COVID-19 vaccines (*6*).

Local and systemic reactions and health impacts reported to v-safe during the week after vaccination were described for children aged 6 months–4 years who received Pfizer-BioNTech vaccine and children aged 6 months–5 years who received Moderna vaccine during June 18–August 21, 2022. VAERS reports were described by serious and nonserious status, demographic characteristics, and MedDRA PTs. Analyses were conducted using SAS software (version 9.4; SAS Institute); p-values <0.05 were considered statistically significant. These surveillance activities were reviewed by CDC and conducted consistent with applicable federal law and CDC policy.^¶¶^

## Review of v-safe Data

During June 18–August 21, 2022, v-safe enrolled 4,749 children aged 6 months–2 years and 3,792 aged 3–4 years who had received Pfizer-BioNTech vaccine and 8,338 children aged 6 months–2 years and 6,387 aged 3–5 years who had received Moderna vaccine (Table 1). Most children (22,695; 97.6%) did not receive any other vaccine at the time of receipt of the first COVID-19 dose. Local and systemic reactions reported during the week after receipt of either Pfizer-BioNTech or Moderna vaccines were most frequently reported on the day after vaccination. Local reactions were reported for 900 (19.0%) children aged 6 months–2 years and 1,078 (28.4%) aged 3–4 years after the first Pfizer-BioNTech vaccine dose and for 1,601 (19.2%) aged 6 months–2 years and 2,072 (32.4%) aged 3–5 years after the first Moderna dose. Systemic reactions were reported for 2,649 (55.8%) children aged 6 months–2 years and for 1,220 (32.2%) children aged 3–4 years after receipt of the first Pfizer-BioNTech vaccine dose and for 4,647 (55.7%) children aged 6 months–2 years and for 2,204 (34.5%) children aged 3–5 years after the first Moderna vaccine dose. The most frequently reported reactions after receipt of either Pfizer-BioNTech and Moderna vaccines among children aged 6 months–2 years were irritability or crying, sleepiness, and fever; among children aged 3–5 years, the most frequently reported reactions were injection site pain, fatigue, and fever. Most reports described reactions as mild to moderate in severity (Table 2).

**TABLE 1:** Adverse reactions and health impacts reported for children aged 6 months–5 years* (N = 23,266) who received Pfizer-BioNTech or Moderna COVID-19 vaccine — United States, June 18–August 21, 2022

Event	Vaccine, age group, % reporting reaction or health impacts after vaccination^†^							
	Pfizer-BioNTech (N = 8,541)				Moderna (N = 14,725)			
	6 mos–2 yrs (n = 4,749)		3–4 yrs (n = 3,792)		6 mos–2 yrs (n = 8,338)		3–5 yrs (n = 6,387)	
	Dose 1 (4,749)	Dose 2 (2,467)	Dose 1 (3,792)	Dose 2 (2,060)	Dose 1 (8,338)	Dose 2 (4,288)	Dose 1 (6,387)	Dose 2 (3,549)
**Any injection site reaction**	**19.0**	**18.3**	**28.4**	**26.5**	**19.2**	**26.7**	**32.4**	**47.1**
Itching	NA	NA	1.9	1.5	NA	NA	1.5	1.7
Pain	13.7	13.3	24.7	23.4	14.2	19.9	29.1	43.5
Redness	5.6	6.3	4.9	5.3	6.1	8.8	4.5	8.1
Swelling or hardness	2.8	1.9	2.2	2.0	2.8	5.7	2.3	4.9
Groin or underarm swelling/tenderness	0.3	0.2	NA	NA	0.4	0.3	NA	NA
**Any systemic reaction**	**55.8**	**47.1**	**32.2**	**29.2**	**55.7**	**58.2**	**34.5**	**49.9**
Abdominal pain	NA	NA	3.5	3.4	NA	NA	4.4	6.3
Myalgia	NA	NA	4.8	3.6	NA	NA	5.0	9.7
Chills	NA	NA	4.0	2.8	NA	NA	3.6	7.7
Fatigue	NA	NA	20.1	19.6	NA	NA	22.9	33.2
Fever	18.7	13.8	12.1	10.9	19.7	27.2	13.5	30.6
Headache	NA	NA	5.0	4.0	NA	NA	5.2	8.7
Joint pain	NA	NA	1.6	0.8	NA	NA	1.0	1.5
Nausea	NA	NA	3.0	2.2	NA	NA	3.0	4.9
Diarrhea	6.7	5.3	4.4	4.0	6.3	5.9	4.3	3.8
Rash	4.9	3.2	2.4	1.4	4.4	3.6	2.2	1.9
Vomiting	3.8	2.8	2.9	2.3	3.6	3.8	3.1	4.1
Irritability/crying	39.6	33.5	NA	NA	39.4	42.7	NA	NA
Loss of appetite	11.7	8.7	NA	NA	10.2	12.9	NA	NA
Sleepiness	25.8	20.9	NA	NA	25.9	28.5	NA	NA
**Any health impact**	**10.3**	**7.5**	**9.3**	**7.4**	**9.8**	**11.6**	**10.8**	**15.9**
Unable to perform normal daily activities	5.3	3.3	5.7	4.1	5.2	6.1	6.6	10.6
Unable to attend child care or school	5.9	4.4	5.6	4.4	5.7	6.5	6.2	7.8
Needed medical care	2.8	2.2	1.7	1.2	2.7	2.4	1.5	1.2
Telehealth	0.8	0.4	0.5	0.3	0.7	0.7	0.5	0.5
Clinic appointment	1.6	1.3	1.0	0.7	1.8	1.5	0.9	0.6
Emergency visit	0.4	0.2	0.2	0.0	0.2	0.1	0.2	0.1
Hospitalization	0.1	0.0	0.0	0.1	0.0	0.0	0.0	0.0

**Abbreviation**: NA = not applicable.

* On June 17, 2022, the Food and Drug Administration amended the Emergency Use Authorizations for BNT162b2 (Pfizer-BioNTech) vaccine to include children aged 6 months**–**4 years and mRNA-1273 (Moderna) vaccine to include children aged 6 months**–**5 years. Safety findings for children aged ≥60 months (5 years) who received Pfizer-BioNTech vaccine have been previously described and were not included in this study.

^†^ Percentage of children whose parents reported a reaction or health impact at least once during days 0–7 post-vaccination. Health check-in surveys were unique for each age group (6 months**–**2 years and ≥3 years).

**TABLE 2:** Most frequent adverse reactions reported to v-safe for children aged 6 months–5 years (N = 23,266)* who received Pfizer-BioNTech or Moderna COVID-19 vaccine, by severity and dose — United States, June 18–August 21, 2022

Event	Age, vaccine, % reporting reaction or health impact after vaccination^†^							
	6 mos–2 yrs (N = 13,087)				3–5 yrs (N = 10,179)			
	Pfizer-BioNTech (n = 4,749)		Moderna (n = 8,338)		Pfizer-BioNTech (n = 3,792)		Moderna (n = 6,387)	
	Dose 1 (4,749)	Dose 2 (2,467)	Dose 1 (8,338)	Dose 2 (4,288)	Dose 1 (3,792)	Dose 2 (2,060)	Dose 1 (6,387)	Dose 2 (3,549)
**Irritability/Crying**	**39.6**	**33.5**	**39.4**	**42.7**	**NA**	**NA**	**NA**	**NA**
Mild	24.4	22.2	25.9	27.9	NA	NA	NA	NA
Moderate	14.5	10.9	12.7	14.1	NA	NA	NA	NA
Severe	0.6	0.5	0.8	0.8	NA	NA	NA	NA
**Sleepiness**	**25.8**	**20.9**	**25.9**	**28.5**	**NA**	**NA**	**NA**	**NA**
Mild	21.2	18.0	21.7	24.5	NA	NA	NA	NA
Moderate	4.4	2.6	3.9	3.9	NA	NA	NA	NA
Severe	0.3	0.2	0.2	0.1	NA	NA	NA	NA
**Injection site pain**	**NA**	**NA**	**NA**	**NA**	**24.7**	**23.4**	**29.1**	**43.5**
Mild	NA	NA	NA	NA	21.4	20.2	25.1	33.1
Moderate	NA	NA	NA	NA	3.0	3.1	3.8	9.8
Severe	NA	NA	NA	NA	0.2	0.1	0.2	0.5
**Fatigue**	**NA**	**NA**	**NA**	**NA**	**20.1**	**19.6**	**22.9**	**33.2**
Mild	NA	NA	NA	NA	11.6	13.2	14.4	19.2
Moderate	NA	NA	NA	NA	7.6	6.2	7.5	12.9
Severe	NA	NA	NA	NA	0.9	0.2	1.0	1.1
**Fever** ^§^	**18.7**	**13.8**	**19.7**	**27.2**	**12.1**	**10.9**	**13.5**	**30.6**
Temperature not documented	6.2	5.1	6.6	7.4	2.7	3.4	2.7	7.2
Temperature documented	12.5	8.8	13.1	19.9	9.4	7.5	10.8	23.4
Normal temperature	4.8	3.9	5.4	7.7	3.5	3.6	3.5	8.6
Documented fever	7.7	4.9	7.7	12.2	5.9	3.9	7.3	14.8
Mild	2.8	1.9	3.0	5.6	2.0	2.0	2.7	7.2
Moderate	2.4	1.8	2.4	3.9	2.0	0.9	2.4	4.3
Severe	2.2	1.0	1.9	2.2	1.6	0.8	1.9	2.9
Very severe	0.3	0.2	0.4	0.5	0.4	0.2	0.3	0.4
**Pain**	**13.7**	**13.3**	**14.2**	**19.9**	**NA**	**NA**	**NA**	**NA**
Mild	12.1	11.9	12.0	16.3	NA	NA	NA	NA
Moderate	1.6	1.3	2.1	3.4	NA	NA	NA	NA
Severe	0.1	0.0	0.1	0.3	NA	NA	NA	NA
**Myalgia**	**NA**	**NA**	**NA**	**NA**	**4.8**	**3.6**	**5.0**	**9.7**
Mild	NA	NA	NA	NA	2.5	2.0	2.7	5.2
Moderate	NA	NA	NA	NA	2.1	1.5	2.1	4.4
Severe	NA	NA	NA	NA	0.2	0.1	0.2	0.2
**Loss of appetite**	**11.7**	**8.7**	**10.2**	**12.9**	**NA**	**NA**	**NA**	**NA**
Mild	6.7	5.4	6.4	8.7	NA	NA	NA	NA
Moderate	4.4	3.0	3.3	3.6	NA	NA	NA	NA
Severe	0.7	0.3	0.5	0.5	NA	NA	NA	NA
**Headache**	**NA**	**NA**	**NA**	**NA**	**5.0**	**4.0**	**5.2**	**8.7**
Mild	NA	NA	NA	NA	3.1	2.9	3.2	5.4
Moderate	NA	NA	NA	NA	1.6	0.9	1.8	3.1
Severe	NA	NA	NA	NA	0.3	0.2	0.3	0.2

**Abbreviation**: NA = not applicable.

* On June 17, 2022, the Food and Drug Administration amended the Emergency Use Authorizations for BNT162b2 (Pfizer-BioNTech) vaccine to include children aged 6 months**–**4 years and mRNA-1273 (Moderna) vaccine to include children aged 6 months**–**5 years. Safety findings for children aged ≥60 months (5 years) who received Pfizer-BioNTech vaccine have been previously described and were not included in this study.

**^†^** Percentage of registrants for whom a parent or guardian reported a reaction or health impact at least once during days 0–7 after vaccination. Includes the most severe event reported during the 0**–**7-day window. Parents and guardians who participate in v-safe use the following definitions to describe the severity of a child’s symptoms: mild (noticeable, but not problematic), moderate (limit normal daily activities), or severe (make daily activities difficult or impossible).

^§^ Fever is self-reported and registrants are not required to record a temperature. Among children who had a reported temperature and met the definition for fever (≥100.4°F [≥38°C]) during days 0–3, fever was classified as mild (100.4°F–101.1°F [38°C–38.4°C]), moderate (101.2°F–102.0°F [38.4⁰C–38.9°C]), severe (102.1°F–104.0°F [38.9°C–40.0°C]), or very severe (>104.0°F [>40°C]).

Parents of approximately 1,323 (5.7% ) and 803 (6.5%) of children aged 6 months–5 years reported that their child was unable to perform normal daily activities in the week after dose 1 and dose 2, respectively of either vaccine. Approximately 741 (2%) reported seeking medical care in the week after either dose; most medical care was received via a clinic appointment (450; 1.3%). Four children received care at a hospital after vaccination; two respondents indicated the hospitalization was unrelated to vaccination, one was unwilling to provide further information, and one completed a VAERS report (Table 3).

**TABLE 3:** Events* reported to the Vaccine Adverse Event Reporting System for children aged 6 months–5 years^†^ after receipt of Pfizer-BioNTech or Moderna COVID-19 vaccine — United States, June 18–August 21, 2022

Adverse events	Vaccine, no. reporting (%)		
	Pfizer-BioNTech	Moderna	Total
**Total**	**496**	**521**	**1,017**
**Vaccination errors**	**278 (56.0)**	**177 (34.0)**	**455 (44.7)**
Error without adverse health event	248 (89.2)	162 (91.5)	**410 (90.1)**
Error with adverse health event**^§^**	30 (10.8)	15 (8.5)	**45 (9.9)**
Error with nonserious health event^¶^	30 (10.8)	14 (7.9)	**44 (9.7)**
Error with serious health event	0 (—)	1 (0.6)	**1 (0.2)**
Nonserious reports (excluding vaccination error MedDRA PTs)**	**486 (98.0)**	**512 (98.3)**	**998 (98.1)**
Fever	84 (17.3)	113 (22.1)	**197 (19.7)**
Rash	52 (10.7)	43 (8.4)	**95 (9.5)**
Vomiting	37 (7.6)	42 (8.2)	**79 (7.9)**
Urticaria	23 (4.7)	43 (8.4)	**66 (6.6)**
Fatigue	29 (6.0)	31 (6.1)	**60 (6.0)**
SARS-CoV-2 negative test result	24 (4.9)	33 (6.5)	**57 (5.7)**
Cough	17 (3.5)	34 (6.6)	**51 (5.1)**
Irritability	16 (3.3)	33 (6.5)	**49 (4.9)**
Decreased appetite	17 (3.5)	29 (5.7)	**46 (4.6)**
Diarrhea	19 (3.9)	26 (5.1)	**45 (4.5)**
Erythematous rash	13 (2.7)	28 (5.5)	**41 (4.1)**
COVID-19	19 (3.9)	18 (3.5)	**37 (3.7)**
SARS-CoV-2 positive test result	18 (3.7)	17 (3.3)	**35 (3.5)**
**Serious reports^††^**	**10 (2.0)**	**9 (1.7)**	**19 (1.9)**
Seizure^§§^	4	3	**7**
Acute left basal ganglia infarction	1	0 (—)	**1**
Acute flaccid myelitis^¶¶^	0 (—)	1	**1**
Anaphylaxis***	0 (—)	1	**1**
Atypical Kawasaki disease	0 (—)	1	**1**
Breath holding	1	0 (—)	**1**
Brief resolved unexplained event	0 (—)	1	**1**
Eye infection with neutropenia	1	0 (—)	**1**
Febrile seizure	1	0 (—)	**1**
Immune thrombocytopenic purpura	1	0 (—)	**1**
Pancreatitis	1	0 (—)	**1**
Tachycardia	0 (—)	1	**1**
Upper respiratory infection with wheezing	0 (—)	1	**1**

**Abbreviations**: MedDRA PT = Medical Dictionary for Regulatory Activities preferred term; VAERS = Vaccine Adverse Event Reporting System.

* Signs and symptoms in VAERS reports are assigned MedDRA PTs by VAERS staff members. Each VAERS report was coded for one or more MedDRA PTs. A MedDRA PT does not represent a medically confirmed diagnosis and might represent a normal finding or a diagnostic test result. Vaccine administration errors that are MedDRA coded are listed separately in this table.

^†^ On June 17, 2022, the Food and Drug Administration amended the Emergency Use Authorizations for BNT162b2 (Pfizer-BioNTech) vaccine to include children aged 6 months**–**4 years and mRNA-1273 (Moderna) vaccine to include children aged 6 months**–**5 years. Safety findings for children aged ≥60 months (5 years) who received Pfizer-BioNTech vaccine have been previously described and were not included in this study.

^§^ The most common MedDRA PTs among reports of vaccination error included incorrect dose administered, product administered to patient of inappropriate age, product preparation issue, wrong product administered, expired product administered, product storage error, and underdose.

^¶^ Adverse health events coded for reports with nonserious vaccination errors included decreased appetite, diarrhea, fatigue, fever, rash, scratch, and vomiting.

** Includes the top 13 most frequently coded MedDRA PTs among nonserious reports.

^††^ Because of the small number of serious reports, percentages are not provided for serious report events. VAERS reports are classified as “serious” only if one of the following events are reported: hospitalization, prolongation of hospitalization, life-threatening illness, permanent disability, congenital anomaly or birth defect, or death. All other reports are classified as “nonserious” (https://www.accessdata.fda.gov/scripts/cdrh/cfdocs/cfcfr/cfrsearch.cfm?fr). Serious reports to VAERS were reviewed by CDC physicians to form a clinical impression, based on available information. In the table, the clinical impression for each report is listed. https://www.meddra.org/how-to-use/basics/hierarchy

^§§^ Six of the seven seizure reports were for afebrile children; temperature was not reported for the other child. Two of the seven reports represented children with preexisting structural brain abnormalities. Three reports of seizures occurring within 24 hours of vaccination were made; one was of an afebrile child with a history of febrile seizures. Two additional reports of seizures were made, occurring 9 days and 18 days after vaccination.

^¶¶^ The acute flaccid myelitis report represented a child recently diagnosed with hand, foot, and mouth disease and human rhinovirus B infection.

*** The anaphylaxis report was for a child who received two vaccinations after a part of the first dose was not injected. Approximately 8 hours after vaccination, the child developed signs and symptoms consistent with anaphylaxis and was treated in an emergency department and discharged.

## Review of VAERS Data

During June 18– August 21, 2022, VAERS received and processed 496 reports of adverse events among children aged 6 months–4 years who had received Pfizer-BioNTech vaccine and 521 reports for children aged 6 months–5 years who had received Moderna vaccine (Table 3).*** Among Pfizer-BioNTech vaccine recipients for whom a VAERS report was submitted, the median age was 3 years, and 249 (50.2%) reports were for events among males. Among Moderna vaccine recipients, the median age was 2 years, and 272 (52.2%) reports were for events among males. Most children (978; 96.2%) for whom reports were submitted received Pfizer-BioNTech or Moderna vaccine as the sole vaccine administered.

Overall, 998 (98.1%) VAERS reports were for nonserious events, including 486 (98.0%) after Pfizer-BioNTech and 512 (98.3%) after Moderna vaccination. The most commonly reported events (455; 44.7%) were related to vaccination errors (e.g., incorrect dose administered, product administered to patient of inappropriate age, or product or preparation issue); among 278 reports of vaccination errors after receipt of Pfizer-BioNTech and 177 reports after receipt of Moderna vaccines, 45 (9.9%) reports indicated that an adverse health event had occurred. Nonserious adverse events most commonly reported were fever (197; 19.8%), rash (95; 9.5%), vomiting (79; 7.9%), urticaria (66; 6.6%), and fatigue (60; 6.0%).

Nineteen serious events were reported to VAERS. Eight reports were for seizure, six of which were reports among children who were afebrile on medical evaluation; one child had a recorded temperature of 102.7°F (39.3°C) and temperature was not reported for the other child. Two children with preexisting diagnoses of structural brain abnormalities experienced seizures in the days after vaccination. One child experienced signs and symptoms consistent with anaphylaxis several hours after vaccination; this child received a partial vaccine dose accompanied by a needle malfunction followed by revaccination with an appropriate dose. No reports of myocarditis after vaccination were reported.

## Discussion

Approximately, one million young children have received an mRNA COVID-19 vaccine. The findings in this report are consistent with those from safety data from preauthorization clinical trials for young children (*3*,*4*). Trial participants aged 6 months–4 years who received Pfizer-BioNTech vaccine and 6 months–5 years who received Moderna vaccine most frequently reported mild or moderate local and systemic reactions; no serious adverse events judged to be related to vaccination were reported in the trial data (*3*,*4*). Initial postauthorization safety monitoring of 19 serious reports identified one report of febrile seizure plausibly associated with vaccination.

Systemic reactions were more frequently reported after COVID-19 vaccination for children aged 6 months–2 years than for children aged 3–5 years. The most frequent reactions reported to v-safe for children aged 6 months–2 years included irritability or crying, sleepiness, and loss of appetite. These reactions are consistent with the clinical trial findings (*3*,*4*) and are common after childhood vaccination (*7*).

Among VAERS reports for Pfizer-BioNTech recipients aged 6 months–4 years and Moderna recipients aged 6 months–5 years, 98% or more were nonserious. Vaccination errors were among the most common events reported to VAERS in this age group. No adverse event was associated with vaccination errors in 92% of these reports. Children in these age groups are authorized to receive a smaller amount of mRNA COVID-19 vaccine than are older children (*8*); incorrect dosing by vaccine administrators in different childhood age groups might lead to vaccination errors. Continued education of vaccine providers might help reduce administration errors, including incorrect dosing, among children. Of the eight seizures reported to VAERS, only one was associated with a fever (39.3°C [102.7°F] ) occurring after COVID-19 vaccination and two were in children with structural brain abnormalities. Myocarditis is a rare adverse event that has been associated with mRNA COVID-19 vaccines; the risk appears highest among adolescents and decreases with decreasing age in childhood (*6*). No events of myocarditis were reported to VAERS after vaccination in children aged 6 months–5 years.

The findings in this report are subject to at least four limitations. First, v-safe is a voluntary program; as a result, v-safe data might not be representative of the vaccinated population. For example, although more doses of Pfizer-BioNTech vaccine than Moderna vaccine were administered to young children in the United States during the surveillance period of this report, more v-safe reports were received for children who received Moderna vaccine. Second, VAERS is a passive reporting system and is subject to reporting biases and underreporting, especially of nonserious events (*5*). Third, Pfizer-BioNTech dose 3 data were not available at the time of this analysis. Finally, these data are limited by the short surveillance period and might change as safety monitoring continues and more doses are administered to children aged 6 months–5 years.

The Advisory Committee on Immunization Practices recommends that all persons aged ≥6 months receive a COVID-19 vaccine (*8*). Initial vaccine safety monitoring in children aged 6 months–5 years are usually similar to those described in clinical trials, and no unexpected safety concerns were detected (*3*,*4*). Health care providers and parents of young children should be advised that local and systemic reactions are expected after vaccination with COVID-19 mRNA vaccines, and serious adverse events are rare. CDC and FDA will continue to monitor vaccine safety and will provide updates as needed to guide COVID-19 vaccination recommendations.

**Summary**

**What is already known about this topic?**

COVID-19 vaccines have been recommended for children aged 6 months–5 years since June 2022; approximately one million doses were administered to persons in this age group during June–August 2022.

**What is added by this report?**

Local and systemic reactions after vaccination with either BNT162b2 (Pfizer-BioNTech) or mRNA-1273 (Moderna) COVID-19 vaccines were reported for children aged 6 months–4 years and 6 months–5 years, respectively, to v-safe and VAERS safety monitoring systems. Serious adverse events were rarely reported.

**What are the implications for public health practice?**

Initial vaccine safety data indicate that among young children, local and systemic reactions are expected after COVID-19 vaccination and serious adverse events are rare.

## Footnotes

* The Pfizer-BioNTech COVID-19 vaccine for use in children aged 6 months–4 years was administered as 3 doses (3 *μ*g [0.2 mL] each), at intervals of 3 weeks between doses 1 and 2 and ≥8 weeks between doses 2 and 3; the Moderna COVID-19 vaccine for use in children aged 6 months–5 years was administered as 2 doses (25 *μ*g [0.25 mL] each), 4 weeks apart. Data for Moderna COVID-19 doses administered to children aged 5 years were unavailable. https://covid.cdc.gov/covid-data-tracker/#vaccination-demographic (Accessed August 16, 2022).

† Children and adolescents aged ≤15 years cannot self-enroll and must be enrolled by a parent or guardian. Health check-ins are sent via text messages that link to web-based surveys on days 0–7 after vaccination; then weekly through 6 weeks after vaccination; and then 3, 6, and 12 months after vaccination.

§ Parents and guardians describe the severity of the child’s symptoms as mild, moderate, or severe. Severity of symptoms for registrants aged ≥3 years is defined as mild (noticeable, but not problematic), moderate (limit normal daily activities), or severe (make daily activities difficult or impossible). The definition of severity of symptoms among registrants aged ≤2 years is unique to each local injection site and systemic reaction. Health impacts among children include inability to perform normal daily activities, missed child care or school, or received care from a medical professional because of new symptoms or conditions.

¶ These reactions were based on data collected in clinical trials and include groin or underarm swelling or tenderness, diarrhea, rash, vomiting, irritability or crying, loss of appetite, and sleepiness.

** Health care providers are required by COVID-19 vaccine EUAs to report certain adverse events after vaccination to VAERS, including death (https://vaers.hhs.gov/faq.html). A VAERS form includes patient information, vaccine information, vaccine administration information, and information regarding the adverse event (https://vaers.hhs.gov/docs/VAERS%202.0_Checklist.pdf).

†† Each VAERS report might be assigned at least one MedDRA PT. A MedDRA coded event does not indicate a medically confirmed diagnosis. https://www.meddra.org/how-to-use/basics/hierarchy

§§ VAERS reports are classified as serious (based on FDA Code of Federal Regulations Title 21) if any of the following are reported: hospitalization, prolongation of hospitalization, life-threatening illness, permanent disability, congenital anomaly or birth defect, or death. https://www.accessdata.fda.gov/scripts/cdrh/cfdocs/cfcfr/cfrsearch.cfm?fr

¶¶ 45 C.F.R. part 46, 21 C.F.R. part 56; 42 U.S.C. Sect. 241(d); 5 U.S.C. Sect. 552a; 44 U.S.C. Sect. 3501 et seq.

*** Processed VAERS reports are those that have been coded using MedDRA, deduplicated, and undergone standard quality assurance and quality control review.
